# The Parental Stress Scale revisited: Rasch-based construct validity for Danish parents of children 2–18 years old with and without behavioral problems

**DOI:** 10.1186/s12955-020-01495-w

**Published:** 2020-08-17

**Authors:** Tine Nielsen, Maiken Pontoppidan, Signe Boe Rayce

**Affiliations:** 1 Department of Psychology, University of Copenhagen, Copenhagen, Denmark; 2 Department of Health, VIVE - The Danish Center for Social Science Research, Copenhagen, Denmark

**Keywords:** Parental stress, Lack of parental satisfaction, Parenting, Construct validity, Rasch model

## Abstract

**Background:**

Experiencing parental stress is common among parents of children of all ages and is elevated in families characterized by stressors such as poverty, mental health problems, and developmental problems. The Parental Stress Scale (PSS) is a short measure for the assessment of perceived stress resulting from being a parent.

**Methods:**

This study examines the construct validity and psychometric properties of the Danish PSS using Rasch and graphical loglinear Rasch models in a sample of parents of 2–18-year-old children with and without known behavior problems. We emphasized analyses of differential item functioning, to ascertain whether the scale yields unbiased scores for subgroups of parents.

**Results:**

The 18-item PSS did not fit the Rasch model or a graphical loglinear Rasch model. After dichotomizing item responses and eliminating items 2 and 11, we found the PSS to consist of two distinct subscales measuring parental stress and lack of parental satisfaction. For the total sample, the Parental Stress subscale fit a very complex graphical loglinear Rasch model with differential item functioning relative to parental education and whether children had behavior problems or not. The Lack of Parental Satisfaction subscale fit a simple graphical loglinear Rasch model with differential item functioning only relative to subsample. When dividing into subsamples of parents of children with and without behavior problems, the Parental Stress subscale fit a simple graphical loglinear Rasch model, though still with differential item functioning, while the Lack of Parental Satisfaction subscale fit the Rasch model in each subsample of parents. Both subscales performed best for parents of children with behavior problems.

**Conclusions:**

The PSS should be used in a 16-item version and scored as two subscales. The PSS appears better suited for use among parents of children with behavior problems than within a sample without any known difficulties.

## Background

Being a parent is both a rewarding and taxing experience, and it is more the rule than the exception for parents to experience parental stress [1–4]. The stress associated with being a parent can be defined as “a set of processes that lead to aversive psychological and physiological reactions arising from attempts to adapt to the demands of parenthood” [5]. Being responsible for the well-being and development of children is demanding, and can, at times, be overwhelming; mainly if parents have limited control over the stressors of everyday life [4]. Parental stress levels are not static and whether or not certain aspects of parenting are experienced as stressful or not depends on the person’s history, their current mental state and well-being and the developmental state of the child [4, 6]. Parental stress can have severe consequences as stress hormones modulate brain function by changing the neuron structure [7]. Whereas short periods of stress can be protective because it prepares the body to respond to acute stress sensibly and effectively, chronic stress can be toxic and have deleterious effects on the body [4, 8]. Bodily changes include imbalances in the neural circuitry in the parent brain [7], and suppression or dysregulation of the immune system [8].

Parental stress is elevated especially among parents experiencing poverty, but is also related to several other factors such as age, gender, temperament, adverse childhood experiences, emotion regulation, coping strategies, social support, loneliness, and mental health problems [4, 9–13]. Some studies find that parental stress decreases as a child grows older [14, 15], whereas more recent research does not find any association between parental stress and child age [16, 17]. The association between parental age and parental stress appear to be curvilinear as very young mothers, and older mothers report higher parental stress levels than mothers aged in between [18, 19]. The relationship between maternal education and parental stress is also curvilinear with both mothers with low education and mothers with high education experiencing higher levels of parental stress than mothers with intermediate education [19–21]. Most studies examine parental stress among mothers only. The evidence on differences in levels of parental stress between mothers and fathers is equivocal as some studies find differences between mothers and fathers, whereas others do not [10]. Thus, fathers may experience parental stress differently than mothers do.

Daily tasks and ordinary aspects of parenting can be stressful for parents of children exhibiting challenging behaviors, and parents of children with developmental disabilities generally have elevated levels of parental stress [22]. This is especially true for the relationship between parental stress and child behavior problems. A recent review shows a relationship between child internalizing behavior and parental stress and an even stronger relationship between child externalizing behavior and parental stress [16]. Most studies in this field are cross-sectional, making it difficult to determine the directionality between parental stress and child behavior [16]. Highly stressed parents tend to employ more harsh and ineffective parenting strategies [23], which may then lead to increased behavior problems, which again increases parental stress [24]. It is possible though that the stress related to caring for a child with developmental problems is qualitatively different from the stress experienced by parents of typically developing children [17].

In order to identify parents who experience high levels of parental stress and offer parent interventions to improve parenting strategies and reduce behavior problems, it is imperative to have a valid and reliable measure of parental stress [24]. Berry and Jones developed the Parental Stress Scale (PSS) as a shorter and less clinical alternative to the Parenting Stress Index (PSI) [25]. The PSS measures “individual differences in the level of stress associated with raising children” [1] and focusses on the individual’s perception of parental stress. The PSS consists of 18 items rated with a 5-point response scale. About half of the items assess pleasurable or positive aspects of parenting, while the rest assess more negative aspects of parenting.

**Fig. 1:**
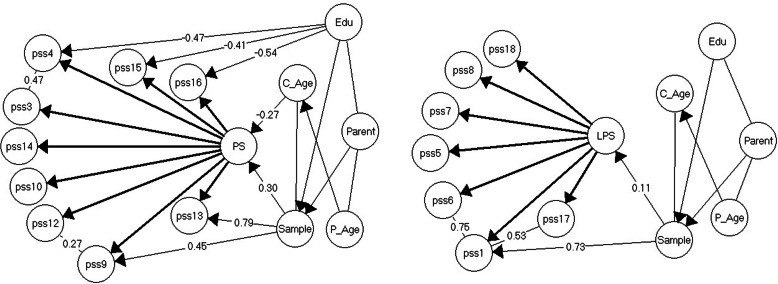
The resulting Graphical loglinear Rasch models for Parental Stress and Lack of Parental Satisfaction subscales for the total sample. Note. γ-correlations are partial Goodman and Kruskal’s rank correlations for ordinal data

Most of the existing validity studies find that the PSS consists of two dimensions characterizing the experience of being a parent; stress and satisfaction, even though they employ different methods and examine different language versions (Additional file 1; Figure 1). In the original development study Berry and Jones [1] used exploratory factor analysis to identify four subdimensions of the PSS falling in the two sub-constructs of parental stress: stress (parental stressors + lack of control) and lack of role satisfaction (parental satisfaction + parental rewards, reversed). Using exploratory methods, Oronoz and colleagues [26] for a Spanish version, Brito and Faro [27] for a Portuguese version, and Cheung [28] for a Chinese version, all propose the same division into the two dimensions as Berry and Jones [1]. In addition, Pontoppidan and colleagues [29], confirmed these two dimensions using Rasch models and a mainly confirmatory approach. Thus, of seven validity studies, five reached the same division of the PSS into two constructs, with the only variation being the number and exact items eliminated during analyses (Additional File 1: Figure 2). The remaining two validity studies both employed confirmatory methods; Algarvio and colleagues used confirmatory factor analysis to test an alternative subscale structure where the PS subscale was divided into three, and the fourth made up by the LPS subscale though with three items excluded [19]. However, they do not present any substantial arguments for this structure and they did not achieve fit to this four-dimensional model. Leung and Tsang [30] analysed the Chinese version using the rating scale model [31], which is simply a Rasch model with the further restriction that the item parameters should be the same for all items (i.e. that the probability of choosing the first response category (and the second, third, and so on) would be the same for all items). The study is the only one, which claim that the PSS is a single construct instrument. However, the claim of fit to the unidimensional model is not a strong claim, as they also report DIF for 6 items, but do not attempt to describe the effect of this DIF or resolve it. In summation, there is compelling evidence across validity studies that item 2 *(There is little or nothing I wouldn’t do for my child(ren) if it was necessary)* should be eliminated, while two studies find that item 11 *Having child(ren) has been a financial burden* should be eliminated and a further two studies find that item 4 (*I sometimes worry whether I am doing enough for my child(ren)*), should be eliminated (Additional file 1: Figure 1). The evidence from previous validity studies also, across varying methods and measurement models, point to the two-dimensional structure of the PSS (Additional file 1: Figure 1).

Positive correlations with the following measures support criterion validity for the PSS: (1) the total score on the Parenting Stress Index [1, 32], (2) depression symptoms measured by the Beck Depression Inventory (BDI) [26, 33], (3) anxiety symptoms measured by the State-Trait Anxiety Inventory [26, 34], (4) the Perceived Stress Scale [1, 35], and (5) parental attitudes (Index of Parent-Child Relations [28, 36]). However, as these studies use different language versions of the PSS with differing numbers of items and measurement properties, and the non-unidimensional structure of at least the BDI and the Perceived Stress Scale is well-documented, they should be compared cautiously.

Only two previous studies have tested the PSS for differential item functioning (DIF), both using Rasch models. Leung and Tsang [30] found that item responses in the total PSS scale did not fit the rating scale version of the Rasch model (c.f. the explanation above) as six items (4, 7, 9, 13, 15, and 16) functioned differentially for parents of primary school children compared to parents of children with ADHD recruited from support groups. The study did, however, not report details on the nature of the bias, nor did the article relay whether (or how) PSS scores were adjusted to account for the differential item functioning. Pontoppidan et al. [29] discovered DIF for two items in the parental stress subscale; DIF relative to age (item 3) and education (items 3 and 16). With the sparseness of investigations for DIF in the PSS, further research is needed on this issue (Additional file 1: Figure 1). Adding to the issue of DIF relative to parent sample discovered by Leung and Tsang [30], previous research on the PSS shows that parental stress levels differ between groups of parents of children with and without developmental difficulties. Studies have found that the PSS is able to differentiate between (1) mothers of children who received treatment for behavioral problems compared to mothers of children not in treatment [1], (2) mothers of children with developmental problems and mothers of children in a non-clinical group [1]; (3) mothers of children with ADHD and mothers of children without ADHD [30]; and (4) mothers of children with parent-child relationship problems and mothers of children without parent-child relationship problems [28]. Depending on the degree of challenges parents face in everyday life with their child, it may be possible that they understand or put different meanings into the items of the PSS. Therefore, in order to study differences in parental stress level, it is imperative to know whether the PSS subscales function equally for these subgroups of parents.

Lastly, only two studies [19, 29] have investigated whether PSS items were more strongly correlated that could be explained by the constructs measured (i.e. items are locally dependent), and both found this not to be the case for items 1 and 17, and one of the studies also for items 3, 4, 9, 10, 12, 16 and 18 (Additional file 1: Figure 1). As unrecognized local dependence between items will artificially inflated reliability estimates, this is a further issue to address for the PSS.

Berry and Jones [1] originally reported that the full PSS had adequate reliability (Cronbach’s alpha = .83) as well as adequate reliability over time (test-retest correlation = .81 over 6 weeks) Other studies [19, 28, 30, 37, 38] have reported corresponding reliability results for different language versions with Cronbach’s alpha ranging from .73 [27] to .90 [30] for the full PSS. The studies that report reliability for the two PSS dimensions separately find varying levels of reliability for both the parental stress dimension (.64–.71 [29]; .76 [26]; and .79 [27]) and the parental satisfaction dimension (.61 [29]; .77 [26]; and .69 [27]). However, as both the language versions, the number of items included, and the samples differ, there are multiple possible causes for the differences in reliability.

In sum, previous validity studies demonstrate that the PSS consists of (at least) two separate unidimensional scales and should not be used as a single scale. In this study, we aim to investigate further the psychometric properties of the Danish PSS using Rasch measurement models in a sample of parents of 2–18-year-old children with and without known behavior problems. We will emphasize the issues of dimensionality and measurement invariance across subgroups of parents and children.

## Methods

### Instrument

The PSS consist of 18 items: 10 items addressing negative and stressful aspects of parenting and eight items addressing positive aspects of parenting [1]. Table 1 present the items of the two proposed subscales. Parents indicate their answer using a 5-point response scale (1 = strongly disagree, 2 = disagree, 3 = undecided, 4 = agree, 5 = strongly agree). The eight positive items are reversed when coding the PSS, and a single parental stress sum score is calculated to indicate the degree of parental stress [1].

**Table 1:** The PSS items divided into the two proposed subscales; Parental Stress and Lack of Parental Satisfaction

Item	**Parental Stress subscale (PS)**
3	Caring for my child(ren) sometimes takes more time and energy than I have to give
4	I sometimes worry whether I am doing enough for my child(ren)
9	The major source of stress in my life is my child(ren)
10	Having child(ren) leaves little time and flexibility in my life
11	Having child(ren) has been a financial burden^a^
12	It is difficult to balance different responsibilities because of my child(ren)
13	The behavior of my child(ren) is often embarrassing or stressful to me
14	If I had it to do over again, I might decide not to have child(ren)
15	I feel overwhelmed by the responsibility of being a parent
16	Having child(ren) has meant having too few choices and too little control over my life
	**Lack of Parental Satisfaction subscale (LPS) – reversely scored items**
1	I am happy in my role as a parent
2	There is little or nothing I wouldn’t do for my child(ren) if it was necessary^a^
5	I feel close to my child(ren)
6	I enjoy spending time with my child(ren)
7	My child(ren) is an important source of affection for me
8	Having child(ren) gives me a more certain and optimistic view for the future
17	I am satisfied as a parent
18	I find my child(ren) enjoyable

^a^items excluded from final models

### Participants and data collection

The total study sample consisted of 805 parents of children aged 2–18 years divided into two subsamples of parents to children *with* known behavior problems and parents to children *without* known behavior problems. The first subsample consisted of data from three intervention studies c: (1) baseline data collected in 2013–2014 from the intervention study Caring in Chaos [39] of which we included 118 parents of children aged 3–9 years old with ADHD symptoms; (2) baseline data collected in 2013–2015 from an intervention study on Parent Management Training Oregon (PMTO) [40] of which we included 108 parents of children aged 4–12 years with behavior problems; (3) baseline data collected in 2018 from another intervention study of PMTO conducted by the Child Centre in Aarhus of which we included eight parents of children aged 5–12 years with behavior problems. The second subsample was collected in 2018 specifically for this study through a targeted Facebook add with a link to a short web survey with a procedure for screening out children with known behavior problems. It consisted of 571 parents with children aged 2–18 In order to facilitate readability of the article, we use the brief name *behavior sample* for the sample of parents to children with known behavior problems and the brief name *ordinary* sample for the sample of parents to children without known behavior problems throughout the article. Table 2 present the demographic characteristics of the study sample.

**Table 2:** Demographic characteristics of the ordinary and behavior samples

	Ordinary sample (*n* = 571)			Behavior sample (*n* = 234)			Total (*n* = 805)		
*Parent*	n	%		n	%		n	%	
Mother	521	91		170	73		691	86	
Father	50	9		64	27		114	14	
*Parent education*	n	%		n	%		n	%	
Secondary or less	55	10		148	63		203	25	
Tertiary	516	90		86	37		602	75	
*Parent Age*	Mean	SD	Range	Mean	SD	Range	Mean	SD	Range
Mother	38.12	6.32	25–55	38.45	5.97	25–56	38.20	6.23	25–56
Father	38.76	5.56	26–52	40.09	6.40	26–55	39,51	6.06	26–55

### Rasch measurement models

The simplest model in the large family of item response theory (IRT) models is the Rasch model (RM) for dichotomous items [41]. In the present study, we used the dichotomous RM, the partial credit model (PCM) [42], which is a generalization of the Rasch model for ordinal data, as well as graphical log-linear Rasch models [43–45]. The dichotomous RM and the PCM generalization adhere to the same requirements for measurement [46, 47]; thus, we hereafter use the term “RM” for the Rasch model. The five basic requirements for measurement of the RM, with the first four providing criterion-related construct validity according to Rosenbaum’s [48] definition, are: 1) *unidimensionality*; the items of a scale measure a single underlying latent construct, 2) *Monotonicity*; the expected item scores increase with increasing values on the latent variable, 3) *Local independence* (or no local dependence; LD); the item responses are conditionally independent given the latent variable, 4) *Absence of differential item functioning* (no DIF); item responses and relevant background variables (i.e., exogenous variables) are conditionally independent given the latent variable, and 5) *Homogeneity*; the rank order of item parameters (item “difficulties”) is the same for all persons regardless their level on the latent variable. Fulfillment of these requirements by a set of items means that the sum score is a sufficient statistic for the estimated person parameter (latent variable). Sufficiency means that no additional information on the latent variable’s score can be obtained from the response profile of the items besides the information provided by the total score. Sufficiency of the raw sum score distinguishes scales fitting Rasch models from scales fitting other IRT models [46]. Sufficiency is desirable when wanting to use the summed raw score of a scale, as it is the case with the PSS. The choice of using the summed raw score or the estimated person parameters (sometimes referred to as the Rasch scores) depends on both the purpose for using the score (i.e., for statistical analysis or individual assessment), the length of the scale, the targeting and the reliability of the scale. One additional factor to take into account concerning the choice of using sum scores or person parameters is the interpretability of these; the first is easily interpreted concerning item scores, while the latter is not as it is a logit scale. These considerations extend to the graphical loglinear Rasch models described below.

When fit to an RM is rejected, it is possible to achieve close to optimal measurement if the only departures from the RM are in the form of uniform differential item functioning (uniform DIF) and/or uniform local dependence (uniform LD) [45]. Uniform/non-uniform refers to the way items depend either on other items or on exogenous variables. Uniform implies that this dependence is the same across all levels of the latent variable, while non-uniform implies that it is not. Uniform DIF or LD can be adjusted for in a graphical loglinear Rasch model (GLLRM), which can be regarded as merely extensions of the RM, allowing precisely these departures from the RM. If a GLLRM is adjusted for uniform LD only, the sufficiency of the sum score is not affected, while the reliability of the scale will be affected to some degree [43–45]. If a GLLRM is adjusted for uniform DIF, the sum score is no longer a sufficient statistic for the latent score, unless the sum score is equated for DIF. The equation for DIF resolves this issue in subsequent comparisons of subgroup scores to avoid confounding by the DIF [49].

#### Item analysis

The strategy of analyses was first to analyze the full 18-PSS with the original five response categories, in order to investigate whether the collapse of response categories conducted by Pontoppidan et al. [29] was also necessary for this sample. As this proved to be the case, we continued the analyses with dichotomous items, again starting with the full 18-item PSS. The analyses rejected the fit to the RM. Therefore, we proceeded with the same overall strategy as Pontoppidan et al. and analyzed each of the subscales made up by the negatively and the reversed positively worded items in the same manner (see Table 1): first, we tested fit of the item responses to the RM. If this was rejected, we proceeded to catalog the departures and subsequently test the fit of the item responses to a GLLRM adjusting for the departures from the Rasch model if these consisted only of uniform LD and/or DIF. When fit to a GLLRM was not achieved, we eliminated the most (statistically and content-wise) problematic item and proceeded again to test fit to the RM and so on.

Overall tests of fit (i.e., global homogeneity by comparison of item parameters in low and high scoring groups) and the overall tests of no DIF were conducted using Andersen’s [50] conditional likelihood ratio test (CLR). The fit of individual items was assessed by conditional infit and outfit statistics [51, 52] and tested by comparing the observed item-restscore correlations with the expected item-restscore correlations under the specified model [44]. The presence of LD and DIF in GLLRMs was tested using two tests [53]; conditional likelihood ratio test of local independence (i.e. no DIF, no LD), and conditional tests of independence using partial Goodman-Kruskal gamma coefficients for the conditional association between item pairs (presence of LD) or between items and exogenous variables (presence of DIF) given the restscores [44]. Evidence of overall fit and no DIF found in the overall tests (CLR) was rejected if this was not supported by individual item fit and lack of evidence of both LD and DIF, in line with the recommendations [54]. Unidimensionality across the PS and LPS subscales was tested by comparing the observed γ correlation of the subscales with the expected γ correlation of the subscales under the unidimensional model, as two subscales measuring different construct will be significantly weaker correlated than what is expected under the unidimensional model [55]. We used the Benjamini-Hochberg procedure to adjust for false discovery rate (FDR) due to multiple testing, whenever appropriate [56]. As recommended by Cox et al. [57], we did not apply a critical limit of 5% for *p*-values as a deterministic decision criterion. Instead, in line with [58], we distinguished between weak to moderate evidence against the model when *p*-values were larger than 0.01, and stronger evidence when *p*-values were less than 0.01.

Reliability was estimated using Hamon and Mesbah’s [59] Monte Carlo method, as this method takes into account any LD in a GLLRM and adjusts the reliability accordingly. Targeting was assessed numerically by two indices as well as graphically. The calculated targeting indices allows for numerical evaluation of targeting [51]. The test information target index is the mean test information divided by the maximum test information for theta, and the root mean squared error (RMSE) target index is the minimum standard error of measurement divided by the mean standard error of measurement for theta. Both indices should preferably have a value close to one. Further, we estimated the target of the observed score and the standard error of measurement (SEM) of the observed score. For a graphical representation of targeting and test information, we plotted items maps with the distribution of the item threshold locations against weighted maximum likelihood estimations of the person parameter locations as well as the person parameters for the population (assuming a normal distribution) and the information function.

#### Exogenous variables

To examine whether DIF was an issue for the PSS, we included five exogenous variables, which have been shown to be associated with parental stress in previous research (c.f. the introduction), or for which previous research have discovered DIF (i.e. parent age and educational level [29, 30];). The resulting five exogenous variables were: Sample, which comprised the parents to children without known behavior problems and parents to children with behavior problems (short names ordinary and behavior samples).

Child age, which was divided into three age groups to obtain adequate groups for DIF-analyses: young children (2–5 years old), children (6–10 years old), and adolescents (11–18 years old). Parent agewas divided into three age categories to get a balanced division (25–35 years, 36–41 years, and 42–56 years), as it was not possible to use the cut point of 30 years, which had been used in previous research for DIF-analysis, due to the distribution in this sample. Parental education was divided into two groups: parents with short education (secondary schooling or less) and parents with long education (tertiary education).

### Software

All analyses were conducted using the DIGRAM software and and item maps were created with R version 3.5.1 [52, 60].

## Results

First, we present the results of the analyses of the total sample of parents including both parents of children with no known behavior issues (named ordinary sample), and parents of children with known/diagnosed behavior problems (named behavior sample). Second, we present results about the separate subsamples.

### Preliminary analyses

In line with the results by Pontoppidan and colleagues [29], we were not able to conduct the analyses using Masters’ [42] partial credit generalization of the RM for ordinal data in the ordinary sample of parents. Instead of improving, the models degenerated, when trying to adjust for departures from the RM. Thus, it would not be possible for us to run the analyses comparing the two parent samples. This was both the main aim of the study and necessary considering the purpose of the instrument; to identify parents in need of attention, support, and intervention. We dichotomized the item responses in the same manner as done in the study by Pontoppidan and colleagues [29], thereby keeping the content and meaning of the response categories; 0 (strongly disagree and disagree) and 1 (undecided, agree and strongly agree), and then proceeded with analyses using the dichotomous RM [41] and GLLRM.

Analysis of the full 18-item PSS showed that it did not fit the RM for the total sample or any of the two subsamples of parents (results not shown), nor was it possible to obtain fit to a GLLRM with all 18 items for any of the samples. In line with Pontoppidan and colleagues (2018), our further analysis showed that many items had to be eliminated in order to establish fit to any model. The remaining items and the discarded items were very similar to the two subscales found in previous studies [1, 26, 28, 29]. To avoid discarding many items to create a unidimensional measure, we proceeded with separate analyses of dichotomized items belonging to the two subscales Parental Stress and Lack of Parental Satisfaction (Table 1).

### The combined total sample (behavior and ordinary samples together)

For the total sample of parents, the proposed 10-item Parental Stress subscale (PS) did not fit the Rasch model, nor did the proposed 8-item Lack of Parental Satisfaction subscale (LPS). Furthermore, it was not possible to establish fit to a GLLRM for any of the two subscales. After eliminating the worst fitting item in each subscale (i.e., LPS item 2 *There is little or nothing I wouldn’t do for my child(ren) if it was necessary* and PS item 11 *Having child(ren) has been a financial burden*, both subscales fitted GLLRMs, though of differing complexity (Fig. 1).

Table 3 presents global tests-of-fit and DIF for the two subscales. We present the item fit statistics in additional files (Additional file 1: Tables 1 and 2).

**Table 3:** Global Tests-of-fit and differential item function for the Parental Stress and the Lack of Parental Satisfaction subscales for the total sample

Tests	PS (RM)			PS (GLLRM)^a^			LPS (RM)			LPS (GLLRM)^b^		
	*CLR*	*Df*	*p*	*CLR*	*df*	*p*	*CLR*	*Df*	*p*	*CLR*	*df*	*p*
Global homogeneity	13.1	8	.11	15.4	15	.43	14.6	6	.02^+++^	10.3	9	.32
*DIF relative to:*												
Child sample	106.0	8	<.0005^+^	16.4	11	.16	44.2	6	<.0005^+++^	22.7	7	<.01^++++^
Children age group	45.5	16	<.0005^+^	47.8	30	.02^++^	42.1	12	<.0005^+++^	33.7	18	.01^++++^
Parent	10.1	8	.26	18.8	15	.22	8.4	6	.21	11.0	9	.27
Parent education	73.6	8	<.0005^+^	17.5	9	.04^++^	17.9	6	<.01^+++^	18.7	9	.03^++++^
Parent age group	22.1	16	.14	51.9	30	<.01^++^	24.5	12	.02^+++^	28.5	18	.05

Notes. *PS* Parental stress, *LPS* Lack of parental satisfaction, *RM* Rasch model, *GLLRM* Graphical loglinear Rasch model, *CLR* Conditional likelihood ratio, *df* degrees of freedom, *p p*-value, *DIF* differential item function

Global homogeneity test compares items parameters in approximately equal-sized groups of high and low scoring parents. The critical limits for the *p*-values after adjusting for false discovery rate were: ^**+**^ 5% limit *p* = .0083, 1% limit *p* = .0017. ^**++**^ 5% limit *p* = .008. ^**+++**^ 5% limit unaltered, 1% limit *p* = .0017. ^**++++**^ 5% limit *p* = .0083, 1% limit *p* = .0017

^a^The GLLRM for the Parental Stress subscale assumed that some items pairs are locally dependent (items 3 and 4, and items 9 and 12), that item 9 and 13 functions differentially relative to child sample, and that item 4, 15 and 16 functions differentially relative to parent educational level

^b^The GLLRM for the Lack of Parental Stress subscale assumed that items 1 and 6, and items 1 and 17 are locally dependent and that item 1 functions differentially relative to child sample

The 7-item LPS subscale fitted a simple GLLRM with strong local dependence between items 17 (*I am satisfied as a parent*) and 1 (*I am happy in my role as a parent*), and items 1 and 6 (*I enjoy spending time with my child(ren)*). Also, item 1 was found to function differentially in relation to the samples, so that parents of children with known behavior problems were systematically *less* likely to agree with the item statement, compared with parents of children with no known behavior issues, no matter their level of lack of parental satisfaction.

The 9-item PS subscale fitted a very complex GLLRM with strong and moderate local dependence between two item pairs as well as varying degrees of DIF for five items. The locally dependent items were: item 3 (*Caring for my child(ren) sometimes takes more time and energy than I have to give*) and 4 (*I sometimes worry whether I am doing enough for my child(ren)*), and item 9 (*The major source of stress in my life is my child(ren)*) and 12 (*It is difficult to balance different responsibilities because of my child(ren)*). We found that two PS items functioned differentially in relation to sample. Parents of children with known behaviour problems were systematically *more* likely to agree with the item statements 9 (*The major source of stress in my life is my child(ren)*) and 13 (*The behavior of my child(ren) is often embarrassing or stressful to me*) than were parents of children with no known behaviour issues, no matter their level of parental stress. Three other PS-items functioned differentially relatively to parents’ educational level. Parents with tertiary education were systematically *more* likely to agree with the item statements 4 (*I sometimes worry whether I am doing enough for my child(ren)*), 15 (*I feel overwhelmed by the responsibility of being a parent*), and 16 (*Having child(ren) has meant having too few choices and too little control over my life*), than parents with secondary or less education, no matter their level of parental stress.

#### The effect of DIF in the PS and LPS subscales

Five items in the PS subscale suffered from DIF; three relative to the educational level of the parents and two relative to sample. In the LPS subscale, one item suffered from DIF relative to sample. To be able to use either the summed scale scores or the estimated person parameters in subsequent statistical analysis or for assessing the parental stress level of individuals, the DIF must be taken into account first by adjusting both scores accordingly. In the additional files, we provide an appendix with conversion tables for this purpose (Additional file 2). These tables provide both the necessary information for converting the summed scale scores to estimated person parameters, the estimated person parameters for all the different subgroups affected by the DIF, and DIF-adjusted scale scores for these subgroups as well (Additional file 2: Table 1 for the PS subscale, and Additional file 2: Table 2 for the LPS subscale). Using the summed and the DIF-equated scores it is possible to investigate to which degree, the DIF would confound any statistical analysis, or possibly lead to erroneous clinical decisions, based on the subscales, if not adjusted for.

Table 4 shows the observed and adjusted mean scores of the PS and LPS for the subgroups affected by DIF in the total sample, as well as the bias (confounding effect) introduced with the use of the unadjusted scores. For the LPS scale, the significant difference in the scores of the two subsamples all but disappeared, when adjusting for the DIF. Failing to adjust the LPS score for DIF would, therefore, lead to an erroneous claim that parents with children with known behavior issues lack parental satisfaction to a significantly higher degree than parents of children without known behavior issues.

**Table 4:** Comparison of observed and DIF-adjusted mean Parental Stress scores and mean Lack of Parental Satisfaction scores in groups affected by differential item functioning in the total sample

DIF-groups (N)	Observed scores		Adjusted scores		
	*Mean*	*SE*	*Mean*	*SE*	Bias^a^
**Parental stress subscale**^**a**^					
*Sample*^*b*^					
Ordinary (571)	4.07	.08	4.45	.08	−.38
Behavior (234)	5.14	.14	4.77	.14	.37
*Parent education*^*c*^					
Secondary or less (203)	4.90	.15	4.52	.15	.38
Tertiary (602)	4.21	.08	4.55	.08	−.34
**Lack of parental satisfaction subscale**					
*Sample*^*d*^					
Ordinary (571)	.69	.04	.69	.04	.00
Behavior (234)	.92	.09	.75	.08	.17

*SE* Standard error

^a^As the Parental Stress subscale is affected by DIF from more than once source, any reference group within a DIF-variable is adjusted to account for the other sources of DIF, and thus all groups are biased if not adjusted

^b^Differences in observed mean scores (*χ*^*2*^ (1) = 45.4, *p* < .001)., and adjusted mean scores (*χ*^*2*^ (1) = 4.1, *p* = .042)

^c^Differences in observed mean scores (*χ*^*2*^ (1) = 15.7, *p* < .001), and adjusted mean scores (*χ*^*2*^ (1) = 0.0, *p* = .849)

^d^Differences in observed mean scores (*χ*^*2*^ (1) = 6.2, *p* = .01), and adjusted mean scores (*χ*^*2*^ (1) = 0.6, *p* = .442)

For the PS subscale, the picture is more complex, because the scores were adjusted for DIF concerning both sample and parent education (Table 4). In terms of making the correct decision when comparing the parental stress scores for subgroups of parents, the adjustments for DIF in both cases meant that the strongly significant difference between the mean scores of parents from each of the two samples was reduced to being marginally significant (*p* = .042), while the strongly significant difference between the mean scores of parents with secondary or less education and parents with tertiary education disappeared. Failure to adjust for DIF would therefore also in this case, lead to a wrong conclusion of differences in the stress levels between parents of children with and without known behavior issues and between parents with different educational levels.

These DIF results are relevant when using the PS and LPS for comparative purposes *between* samples of parents to children with known behavior problems and parents to children without known behavior problems (i.e., research purpose).

### The behavior and ordinary samples separately

To facilitate use *within* the two populations represented by the two samples, for the purpose of fair assessment of individual parents in relation to the relevant child context, we conducted a separate set of analyses for each of the samples.

**Fig. 2:**
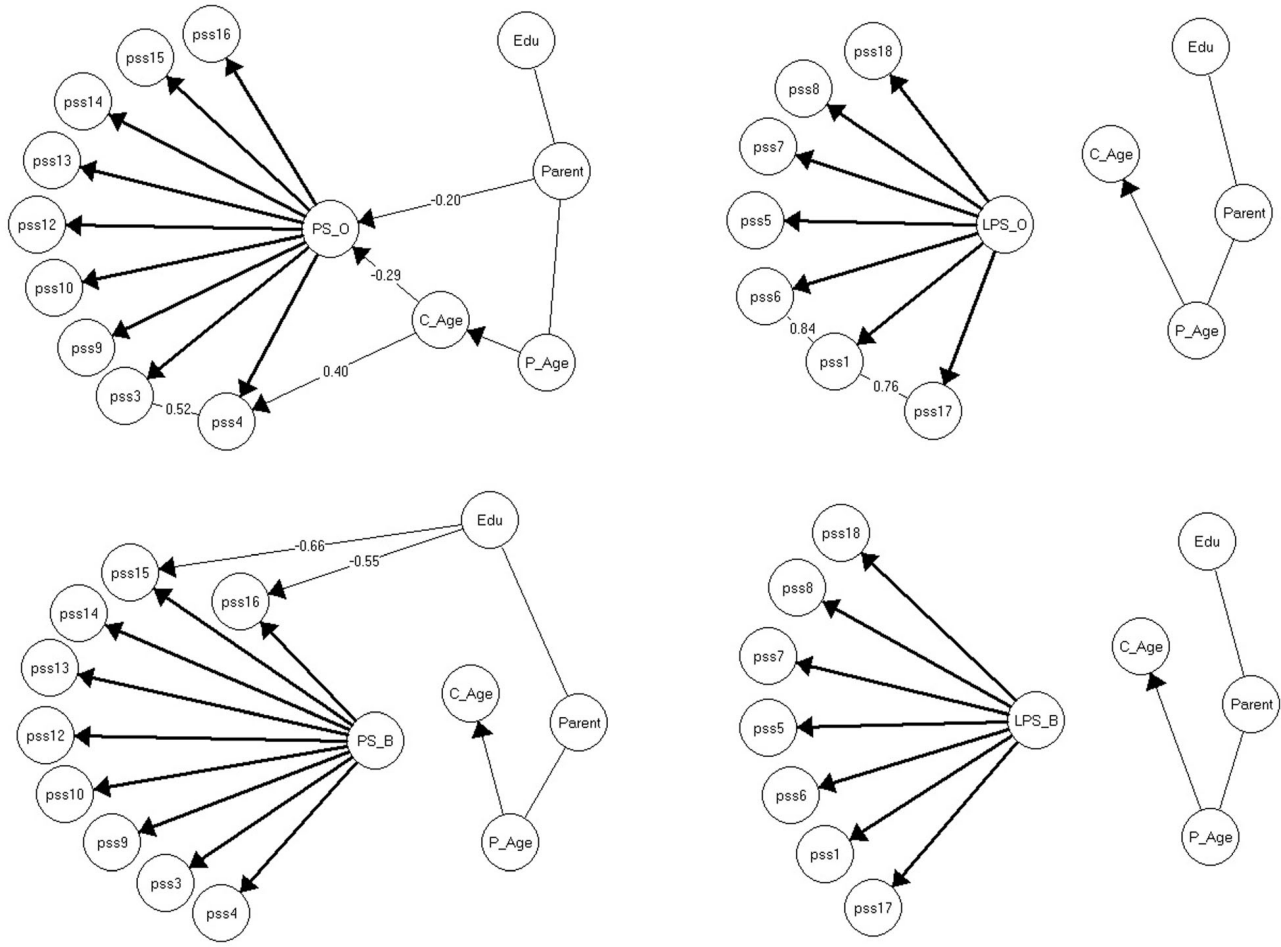
The resulting Graphical loglinear Rasch models and Rasch model for the Parental Stress subscale and the Lack of Parental Satisfaction subscale in the behavior sample (top) and ordinary sample (bottom). Note. γ-correlations are partial Goodman and Kruskal’s rank correlations for ordinal data

For the behavior sample, the PS and LPS scales each fitted simpler models compared to the combined total sample. For the ordinary sample, the PS scale likewise fitted a simpler GLLRM, while the model for the LPS scale was identical to the model for the combined total sample. One reason for the simpler models was that splitting the total sample eliminated the sample-DIF. However, the separate analyses for the behavior and ordinary samples also allowed us to ascertain the differences in the functioning of the scales in the two samples in more detail (Fig. 2).

The PS subscale fitted simple GLLRMs in each of the two samples. For the behavior sample, the GLLRM included DIF relative to the educational level of the parents for two items. Parents with tertiary education were systematically *more* likely to agree on the statements of item 15 and 16 than were parents with secondary or less education, no matter their level of parental stress. While we found DIF in relation to item 4 for the combined total sample, we found no DIF in relation to this item in the behavior sample. Furthermore, the GLLRM for the behavior sample did not include any LD between items either. In the ordinary sample, the GLLRM for the PS subscale included local dependence between items 3 and 4, while there was no evidence of local dependence between items 9 and 12 as found for the combined total sample. We found evidence of DIF for item 4 relative to child age for the ordinary sample so that with increased child age the parents were *more* likely to agree with the item statement, no matter their level of parental stress.

The LPS subscale fitted a pure Rasch model in the behavior sample, and a very simple GLLRM in the ordinary sample; we found no evidence of DIF, and only local dependence between items 17 and 1, and 1 and 6.

Table 5 present global tests-of-fit and DIF for the final subscale model in each of the parent samples. Item fit statistics are provided in the Additional file 1: Tables 3 and 4.

**Table 5:** Global Tests-of-fit and differential item function for the final models of the Parental Stress and the Lack of Parental Satisfaction subscales to the models for the separate behaviour and ordinary samples depicted in Fig. 2

Tests	Behaviour sample						Ordinary sample					
	PS (GLLRM)^a^			LPS (RM)			PS (GLLRM)^b^			LPS (GLLRM)^c^		
	*CLR*	*df*	*p*	*CLR*	*df*	*P*	*CLR*	*Df*	*p*	*CLR*	*df*	*p*
Global homogeneity	5.0	10	.89	8.1	6	.23	17.2	11	.10	1.8	8	.99
*DIF relative to:*												
Children age group	27.2	20	.13	8.5	12	.74	25.2	16	.07	26.2	16	.05
Parent	16.8	10	.08	3.3	6	.77	7.1	11	.80	4.0	8	.85
Parent education	6.6	6	.36	2.6	6	.85	23.3	11	.02^**+**^	11.3	8	.18
Parent age groups	31.1	20	.05	14.3	12	.29	24.5	22	.32	19.1	16	.26

Notes. *PS* Parental stress, *LPS* Lack of parental satisfaction, *RM* Rasch model, *GLLRM* Graphical loglinear Rasch model, *CLR* Conditional likelihood ratio, *df* degrees of freedom, *p p*-value, *DIF* differential item function

Global homogeneity test compares items parameters in approximately equal-sized groups of high and low scoring parents

^+^ The critical 5% limit for the p-values after adjusting for FDR was *p* = .0100

^a^The GLLRM for the PS scale assumed that item 15 and 16 functions differentially relative to parent educational level

^b^The GLLRM for the PS scale assumed that items 3 and 4 are locally dependent, and that item 4 functions differentially relative to child age

^c^The GLLRM for the LPS subscale assumed that items 1 and 6, and item 1 and 17 are locally dependent

#### The effect of within-sample DIF in the PS and LPS subscales

Additional file 2 Tables 3-6 provide conversion tables, information for adjusting both sum scores and person parameters for the DIF in the parental stress scale for each sample. Adjusting the PS sum score for DIF within each of the samples did not alter the conclusion regarding group differences (Table 6).

**Table 6:** Comparison of observed and equated mean Parental Stress scores in parent education groups for the behavior sample and child age groups for the ordinary sample

Samples and subgroups (n)	Observed scores		Adjusted scores		Bias
	*Mean*	*SE*	*Mean*	*SE*	
**Behavior sample**					
*Parent education*^*a*^					
Secondary or less (148)	5.10	.17	5.10	.17	.00
Tertiary (86)	5.21	.22	5.63	.23	−.42
**Ordinary sample**					
*Child age groups*^*b*^					
2–5 years (174)	4.75	.15	4.75	.15	.00
6–10 years (201)	4.18	.13	4.10	.13	.09
11–18 years (196)	3.35	.14	3.14	.14	.22

*SE* Standard error.

^a^Differences in observed mean scores (*χ*^*2*^ (1) = 0.2, *p* = 0.696), and adjusted mean scores (*χ*^*2*^ (1) = 3.5, *p* = 0.062)

^b^Differences in observed mean scores (*χ*^*2*^ (2) = 48.7, *p* < .001), and adjusted mean scores (*χ*^*2*^ (2) = 61.8, *p* < .001)

#### Targeting and reliability of the PS and LPS subscales

Targeting of the PS and LPS subscales were very different (Table 7).

**Table 7:** Targeting and reliability of the Parental Stress and Lack of Parental Satisfaction subscales in the separate behaviour and ordinary samples

	Theta								Score			
Subscales, Samples and Parent subgroups^a^ (n)	Target	Mean	TI mean	TI max	TI Target index	RMSE mean	RMSE min	RMSE target index	Target	Mean	Mean SEM	r
**Parental Stress subscale**												
*Ordinary sample*												
Child age 2–5 years (174)	−.95	−.03	1.318	1.465	.900	.865	.826	.955	3.45	4.75	1.14	.74
Child age 6–10 years (201)	−.19	−.50	1.322	1.382	.956	.861	.851	.988	4.63	4.18	1.15	.70
Child age 11–18 years (196)	.07	−.1.25	1.196	1.341	.892	.884	.863	.977	5.02	3.35	1.09	.72
*Behavior sample*												
Secondary edu or less (148)	−.30	.36	1.286	1.556	.826	.892	.802	.899	4.21	5.10	1.13	.71
Tertiary edu (86)	−.58	.86	1.124	1.341	.839	.930	.864	.929	3.50	5.21	1.06	.72
**Lack of Parental Satisfaction subscale**												
Ordinary sample (571)	−.92	−3.91	.430	1.706	.252	1.320	.766	.580	3.08	.69	.60	.55
Behavior sample (234)	.32	−3.14	.518	1.367	.379	1.289	.855	.663	3.85	.92	.65	.71

Notes. *TI* Test information, *RMSE* The root mean squared error of the estimated theta score, *SEM* The standard error of measurement of the observed score, *r* reliability, *Edu* Education

^a^Targeting and reliability is provided for groups defined by DIF variables

**Fig. 3:**
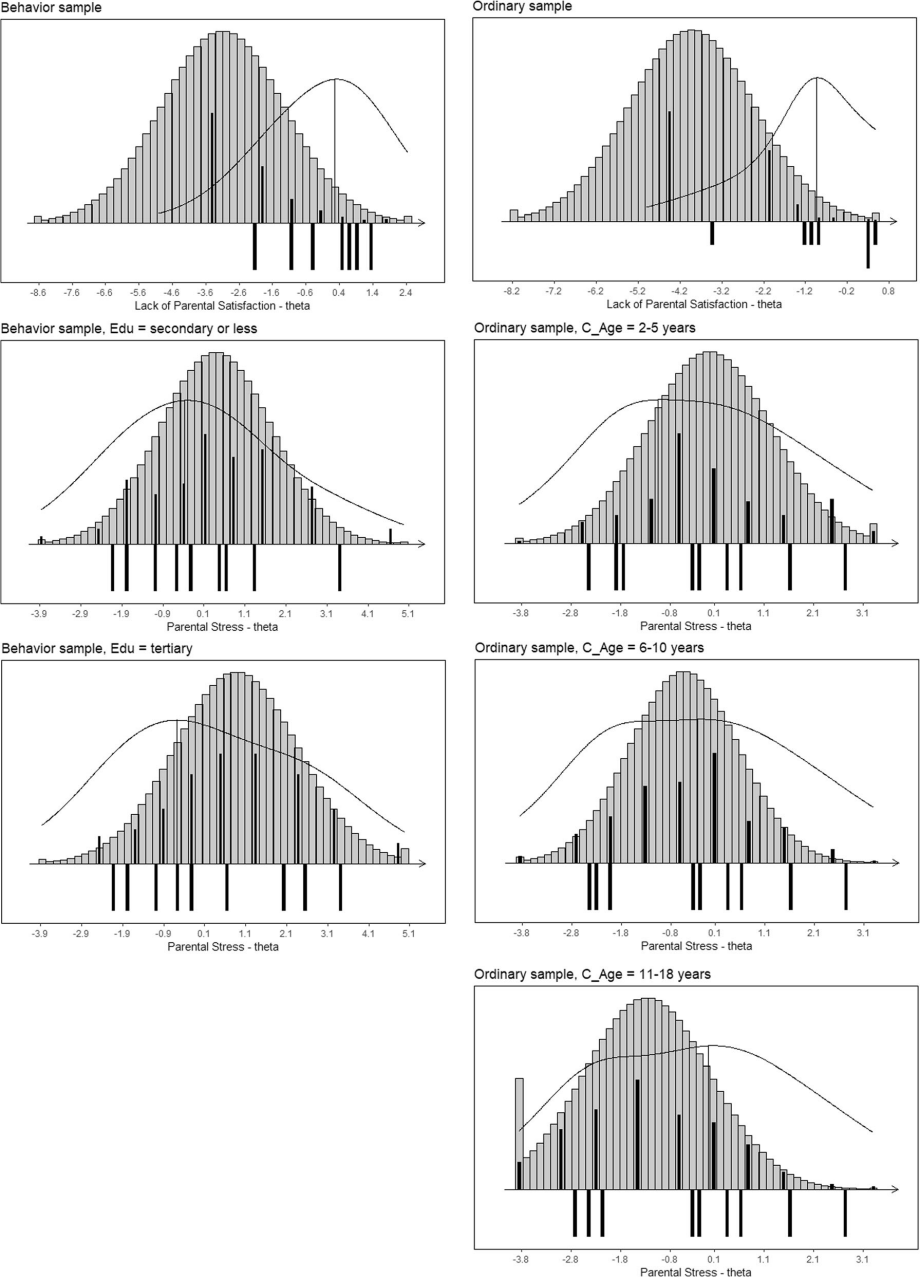
Item maps with distributions of person parameter locations and information curve above item threshold locations. Notes. Person parameters are weighted maximum likelihood estimates and illustrate the distribution of these for the study sample (black bars above the line) and for the population under the assumption of normality (grey bars above the line), as well as the information curve, relative to the distribution of the item difficulties (black bars below the line). For the PS subscale, item maps are shown in subgroups for which evidence of DIF was found within each of the two samples

The targeting of the LPS subscale was very poor for both the behavior and ordinary sample (38 and 25% of the maximum information was achieved, respectively). This very poor targeting is a result of the person parameters for the majority of the parents being located at the low end of the LPS scale (e.g., they did not lack parental satisfaction), while the item parameters (e.g., the item difficulties denoting how lacking a parent should be in parental satisfaction in order to endorse the different items), as well as the area with most information, were located more toward the high end of the LPS scale (Fig. 3).

Targeting of the PS subscale was excellent in both the behavior and ordinary sample (i.e., parents of children with and without known behavior issues, respectively). In the behavior sample, targeting did not differ for groups of parents with secondary or less education compared to parent with tertiary education (with 83 and 84% of the maximum information obtained). In the ordinary sample, targeting differed slightly across groups of parents defined by child age, with the absolute best targeting for parents of children aged 6–10 years (96% of the maximum obtainable information). This excellent targeting is also illustrated by person parameters for the parents being quite symmetrically in relation to the item parameters along the latent PS scale, and that most person parameters are within the area with the most information (Fig. 3).

The reliability of the PS subscale was above .70 for both the ordinary and the behavior sample. This includes the subgroups of parents for which the scale functioned differentially (Table 7). Thus, by conventional standards, the reliability of the PS subscale was at an acceptable level for all parents included in the study, and the reliability of the LPS subscale was at an acceptable level for the behavior sample (.71), but not for the ordinary sample (.55).

### Dimensionality and subscale correlation

We formally tested whether the PS and the LPS subscales made up a single unidimensional scale, both with the total sample and divided into the ordinary and behavior samples, as amount of local dependence in the subscale models differed across the samples (Fig. 1 and Fig. 2). In all three samples the observed correlation between the PS and LPS subscales was weaker than the expected correlation under a unidimensional model (total sample: γ_observed_ = 0.306, γ_expected_ = 0.503, SE = 0.033, *p* < 0.001; ordinary sample: γ_observed_ = 0.262, γ_expected_ = 0.487, SE = 0.042, *p* < 0.001; behavior sample: γ_observed_ = 0.374, γ_expected_ = 0.567, SE = 0.052, *p* < 0.001), and thus the unidimensionality of the full PSS was rejected in all three cases.

In addition, the observed correlation between the PS and the LPS subscales was strong in the sample of parents to children with behavior problems (the behavior sample), while only moderate in the sample of parent to children without known behavior problems (the ordinary sample).

## Discussion

In this study, we examined the psychometric properties of the 18-item PSS among parents of 2–18 year-olds using RM and GLLRM. We furthermore tested the psychometric properties in two subsamples: a sample of parents of children with known behavior problems (named the behavior sample) and a sample of parents of children without known behavior problems (named the ordinary sample). After dichotomizing responses and eliminating items 2 and 11, we found that the PSS comprises two separate unidimensional subscales: parental stress (PS) and lack of parental satisfaction (LPS). This is in accordance with findings in several other national settings [1, 26–28] as well as in a the study among Danish mothers of infants using RM and GLLRM [29]. We further found that in the total sample, both subscales presented local dependency and differential item functioning but fitted a GLLRM after DIF and LD had been taken into account. Divided into the two parent samples, the LPS subscale fitted a pure Rasch model in the behavior sample and a simpler GLLRM with LD but no DIF in the ordinary sample. The PS subscale suffered from DIF in the behavior sample and both LD and DIF in the ordinary sample but fitted a GLLRM in both samples after LD and DIF had been adjusted for. Finally, the two subscales performed best for parents of children with behavior problems. Measures like the PSS are constructed primarily to aid identification and monitor progress in families that feel challenged and stressed as parents. The fact that we find the psychometrically most parsimonious models for the behavior sample, is therefore positive. Thus, our findings support the construct validity of the parental stress construct when operationalized as two separate subscales (parental stress and lack of parental stress).

The elimination of the LPS item 2 *There is little or nothing I wouldn’t do for my child(ren) if it was necessary* is in accordance with all previous studies [1, 19, 26–30]. The elimination of the PS item 11 *Having child(ren) has been a financial burden* is consistent with the original study [1] and the previous Danish study comprising mothers of infants [29], but is not supported in the remaining validity studies. As the validity studies are conducted in countries with very different levels of economic support for families, this item may be particularly prone to cultural differences, and it is essential to be aware of this when using the PSS in different contexts in the future. Furthermore, the various validity studies eliminate between two and five items, and different ones, thus the scores resulting from any two validity studies are comparable, except for the present study and the study by Pontoppidan and colleagues [29] on another Danish sample, where items were also dichotomized.

### Sample DIF

In the total sample, we found DIF relative to parent sample for both the PS and the LPS. For the LPS item 1, parents of children with known behavior problems were systematically *less* likely to agree with the item statement, compared with parents of children with no known behavior issues, no matter their score, whereas the opposite was found for PS items 9 and 13. For the PS subscale, using the DIF-adjusted scores did not alter the conclusion that parental stress was significantly higher in for parents to children with behavior problems. For the LPS subscale, using the DIF-adjusted scores revealed that there was no significant difference in the lack of parental satisfaction of parents in the two samples, as was suggested if scores were not adjusted.. This result highlights that failing to adjust for sample DIF may lead to false conclusions on differences between the sample subgroups of parents. Children with behavior problems often show externalizing behavior and a high level of conflict between parent and child. It is therefore not surprising that parents of children with behavior problems are systematically less likely to agree item 1 “*I am happy in my role as a parent*”, and more likely to agree on item 9 ‘*The major source of stress in my life is my child(ren)’* and item 13 ‘*The behavior of my child(ren) is often embarrassing or stressful to me.’* Previous studies have found, that mothers of children with behavioral problems [1] or ADHD [30] show higher levels of parental stress measured by the PSS compared to mothers of children without behavioral problems or ADHD. As one study did not examine DIF, and it is unclear whether the other adjusted for it, these differences may be due to DIF and not genuine differences in the stress and lack of parental satisfaction of between parents to children with and without know behavior problems..

### DIF relative to parental education

For the total sample three items (4, 15 and 16) in the PS subscale showed DIF relative to the level of parental education, such DIF was only present for two items (15 and 16) in the behavior sample, but not in the ordinary sample. In the total sample, failing to adjust for DIF relative to parental educational level would lead to a false conclusion on parents with a lower education, having an artificially higher level of parental stress. In the behavior sample, the consequences of failing to adjust for DIF were less pronounced. However, bearing in mind the relatively small sample of parents with children with known behavior problems, the difference of half a point between the parents with a low and a high level of education would become significant even with a modest increase in sample size. A previous study examining the PSS in a sample of 3842 Portuguese parents of children 3–10 years old found that mothers and fathers with lower levels of education reported higher levels of parental stress measured by the PSS [19]. As they did not examine or adjust for DIF, it is possible that also these differences are due to DIF and not real differences in stress levels for parents with differing educational levels.

### DIF relative to child age

DIF in relation to child age was only discovered for item 4 (*I sometimes worry whether I am doing enough for my child(ren)* in the PS subscale for the ordinary sample. Using DIF-adjusted scores parents of younger children reported significantly higher levels of parental stress compared to parents of older children.. Some previous studies find results supporting this finding [14, 15], whereas other studies do not find any association between parental stress and child age [16, 17]. It is, however, difficult to examine the effect of child age on the level of parental stress as many families have more than one child adding to the total number of stressors on the family, but it has been found that parental stress increase with the number of children [19]. This is likely so, because it is costlier both economically, emotionally, and time-wise to have more children. Future research into parental stress would benefit from employing a design allowing to evaluate the effect of number of children and their age levels, as well as investigating the potential interaction-DIF between these.

### Reliability and targeting

With a single exception (the LPS subscale in the ordinary sample), the reliability of the PS and the LPS subscales was at an acceptable level (above .70) for use in large scale surveys in all subgroups of parents. The low reliability was most likely caused by less variation in this sample compared to the behavior sample. Another validity study of the Danish PSS [29], although conducted in a community sample of mothers to infants aged 0–12 months, also found the LPS subscale to have the lowest reliability. Relatively low reliability is of less concern in large samples where the risk of type 2 errors is smaller. The reliability of the PS and LPS subscales do, however, not meet the .90 that is usually recommended for a screening tool in either of the samples in this study. As reliabilities are not at the level required for diagnostic tests, we foremost recommend that the PSS is used in large-scale surveys. However, if the suboptimal reliability and the margin of error this causes is kept in mind, the PS subscale and the LPS subscale (if used among parents to children with behavioral problems) may also be used cautiously for screening purposes. This should, however, be done keeping in mind that further research and application tools such as conversion tables are needed.

The targeting of the PS subscale was excellent in both samples, whereas the targeting of the LPS subscale was very poor. The poor targeting of the LPS subscale indicates that precision is not optimal when measuring lack of parental satisfaction for the groups of parents used in this study. This finding is similar to a previous study [29] and refers to the fact that most fathers and mothers find it rewarding and satisfying to be a parent – also parents of children with behavioral problems. We recommend that future studies include in families with a broader range of challenges such as poverty, mental health issues, and substance abuse, as a targeting might be better for such groups.

### Strengths and limitations

A strength of the current study is our use of graphical loglinear Rasch model to adjust for bias caused by DIF, when this was found to be a cause of the lack of fit to the Rasch model, rather than simply discarding such items. Our use of these models also allowed us to take into account any lack of local independence between items, when calculating reliability, thereby avoiding inflation of this. Another strength is the inclusion of fathers in our sample, as most previous studies have mostly included mothers. Similarly, the large age span of the children of the parents is considered a strength because it allowed us to test for DIF relative to the child age. Finally, it is a strength that we included both parents of children with and without known behavior problems, and that we were able to test whether the instrument functioned equally well across these parent groups. A limitation of the study is that the sample is relatively small. However, as this is a first validity study including both mothers and fathers of children with and without known behavior problems, our study does pave the way for studies with larger and more diverse samples in terms of psychological, physical and social issues of the children. Another limitation is that we were not able to obtain information on the number of children in the household for all parents.

Despite the limitations of the current study, it expands the findings of Pontoppidan et al. [29] substantially, by providing sound results on the validity of the PSS for both mothers and fathers, parents of children aged 2–18, and parents of children with or without known behavior issues. The psychometric properties of the Danish PSS are thus available for mothers of infants (aged 0–12 months) and children (aged 2–18 years) with and without known behavior issues. To facilitate a complete package of conversion tables for practitioners and researchers, we recommend that future studies are undertaken to fill the gaps; including mothers and fathers of children with and without known behavior issues aged 1–2 years, as well as a study including fathers of infants. We further suggest that psychometric studies are also undertaken in samples of parent to children with other types of difficulties and disorders, and preferably compared to parents of children with no known difficulties, so that unbiased comparisons are facilitated. In the present study, it was necessary to dichotomize the response categories in order to make the PS and LPS subscale fit an RM or GLLRM in the ordinary sample, thereby making a comparison between the two subsamples possible. This may, however, not be necessary if PSS is administered in a more clinical sample. We therefore also suggest that further psychometric studies are conducted to test whether the PSS can be scored using the original five response categories when used in samples that are more clinical.

## Conclusions

In conclusion, we recommend that the (The Danish) PSS is administered in the 16-item version, excluding items 2 and 11, with questions in the original order. Further, in line with Pontoppidan et al. [29], we recommend using the original response categories to meet respondents need to differentiate their answer, and to apply the dichotomization of the response categories as well as the division into parental stress and lack of parental satisfaction subscales in the scoring and interpretation procedure.

## Supplementary information

**Additional file 1.** Contains a figure with the subscale structure of the PSS in previous validity studies and four tables with item fit statistics.

**Additional file 2.** Appendix with conversion tables.

**Additional file 3.** Contains consistency tables with inter-item correlations and item-restscore correlations for each subscale in each subsample.

## Data Availability

The datasets generated and analyzed during the current study are not publicly available to protect patient privacy but are available from the corresponding author on reasonable request.

## Abbreviations

PSS: Parental Stress Scale
PSI: Parenting Stress Index
BDI: Beck Depression Inventory
ADHD: Attention deficit hyperactivity disorder
PS: Parental stress
LPS: Lack of parental satisfaction
PMTO: Parent Management Training Oregon
IRT: Item response theory
RM: Rasch model
PCM: Partial credit model
LD: Local dependence
DIF: Differential item functioning
GLLRM: Graphical loglinear Rasch model
CLR: Conditional likelihood ratio
RMSE: Root mean squared error
SE: Standard error
