# Supplementary material for: The Parental Stress Scale revisited: Rasch-based construct validity for Danish parents of children 2–18 years old with and without behavioral problems
Source: Health Qual Life Outcomes. 2020 Aug 17;18:281. doi: 10.1186/s12955-020-01495-w (PMC7430114; doi:10.1186/s12955-020-01495-w)
Supplement: Supplementary file 1 — Additional file 1. Contains a figure with the subscale structure of the PSS in previous validity studies and four tables with item fit statistics. [file 12955_2020_1495_MOESM1_ESM.pdf]

Additional file 1:

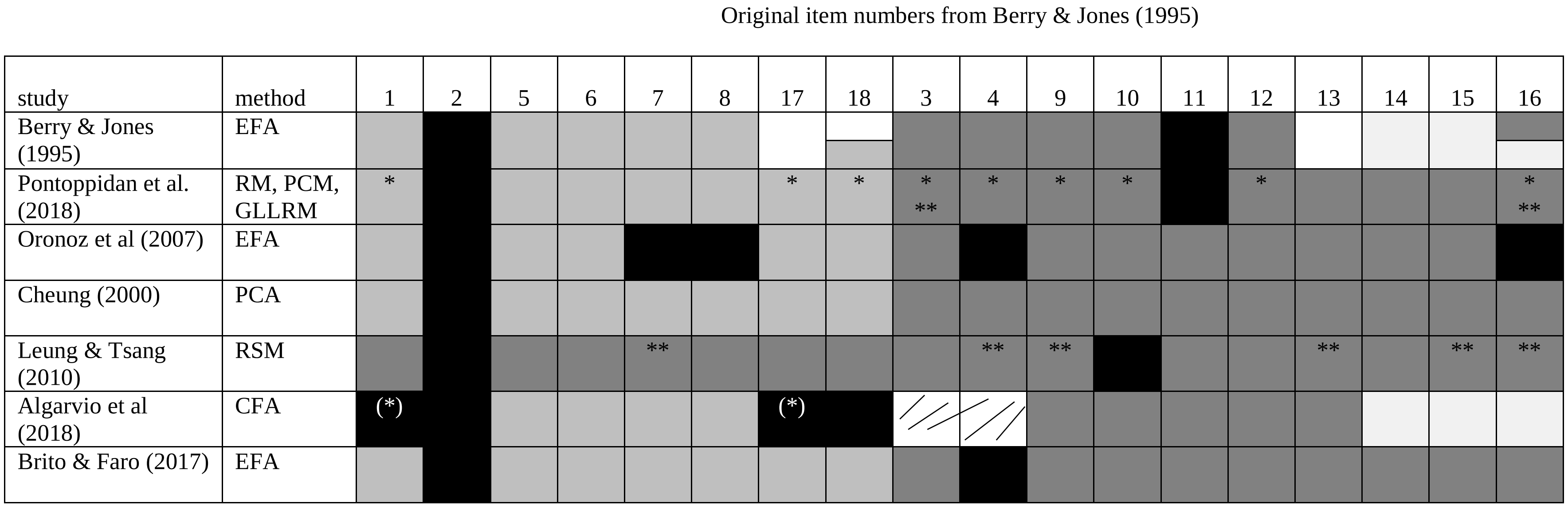

Additional file 1: Figure 1 Division of PSS items into factors and/or subscales in six previous studies related to the structure in the original development study by Berry & Jones (1995)

Notes. EFA = Exploratory factor analysis, RM = Rasch model, PCM = partial credit model, GLLRM = graphical loglinear Rasch model, PCA = principle component analysis, RSM = rating scale model, CFA = confirmatory factor analysis, * = locally dependent items (in studies where no items are marked this has not been investigated), (*) = locally dependent items, which were eliminated, ** = DIF items (in studies where no items are marked this has not been investigated). Subscale colouring takes departure in the four factors in Berry and Jones (1995) development study (colour legend below), with factors/subscales in the other studies coloured accordingly.

Colour legend:

= parental stressors
= lack of control
= parental satisfaction
= parental rewards
= item eliminated
= additional and unique subdivision in Algarvio et al (2018)

Additional file 1: Table 1 Item fit statistics for the Parental Stress subscale under the RM and the GLLRM for the combined total sample

Model & items	outfit	*p*	infit	*p*	Observed γ	Expected γ	*p*
Rasch model							
3	0.769	0.212	0.849	0.014^{+}	0.668	0.562	0.017^{+}
4	1.230	0.310	1.045	0.517	0.503	0.568	0.181
9	0.901	0.250	0.912	0.031^{+}	0.606	0.538	0.065
10	1.327	0.081	1.107	0.062	0.498	0.562	0.157
12	0.850	0.082	0.923	0.060	0.600	0.539	0.102
13	1.000	0.287	1.051	0.238	0.507	0.544	0.334
14	1.593	0.096	1.023	0.844	0.549	0.625	0.231
15	1.291	0.004^{+}	1.103	0.050	0.461	0.549	0.023^{+}
16	0.962	0.823	0.889	0.093	0.642	0.586	0.209
Graphical loglinear Rasch model[a]							
3	0.793	0.274	0.890	0.101	0.668	0.594	0.093
4	1.451	0.086	1.150	0.050	0.503	0.624	0.009^{++}
9	0.999	0.994	0.972	0.539	0.606	0.585	0.544
10	1.146	0.381	1.006	0.917	0.498	0.497	0.983
12	0.867	0.130	0.940	0.163	0.600	0.554	0.214
13	1.010	0.896	1.029	0.516	0.507	0.523	0.692
14	1.350	0.241	1.005	0.966	0.549	0.590	0.546
15	1.182	0.039^{++}	1.069	0.123	0.461	0.522	0.128
16	0.902	0.509	0.884	0.087	0.642	0.574	0.139

Notes. γ = Goodman & Kruskal's gamma coefficients. RM: Rasch model; GLLRM: Graphical loglinear Rasch model

The critical limits for the p-values after adjusting for false discovery rate were: $^{+}$ 5% limit p = .00185, $^{++}$ 5% limit p = .00172, thus no items showed significant misfit.

[a] The GLLRM for the Parental Stress subscale assumed that some items pairs are locally dependent (items 3 and 4, and items 9 and 12), that item 9 and 13 functions differentially relative to child sample, that item 4, 15 and 16 functions differentially relative to parent educational level.

Additional file 1: Table 2 Item fit statistics for the Lack of Parental Satisfaction subscale under the RM and the GLLRM for the total sample

Model & items	Outfit	*p*	infit	*P*	Observed γ	Expected γ	*p*
Rasch model							
1	0.889	0.455	0.860	0.140	0.721	0.675	0.448
5	1.023	0.935	1.144	0.309	0.718	0.743	0.727
6	0.462	0.063	0.809	0.189	0.892	0.747	0.049

7	1.372	0.240	1.281	0.068	0.604	0.754	0.050
8	1.252	0.128	1.036	0.669	0.538	0.579	0.466
17	0.845	0.246	0.897	0.250	0.725	0.661	0.278
18	1.372	0.352	0.958	0.818	0.694	0.768	0.391
Graphical loglinear Rasch model[a]							
1	1.150	0.458	1.040	0.726	0.721	0.772	0.297
5	0.822	0.457	0.995	0.971	0.718	0.648	0.420
6	0.627	0.344	1.027	0.868	0.892	0.852	0.458
7	1.045	0.867	1.096	0.530	0.604	0.658	0.564
8	1.047	0.717	1.004	0.964	0.538	0.547	0.877
17	0.916	0.568	0.952	0.605	0.725	0.697	0.611
18	1.045	0.894	0.895	0.565	0.694	0.670	0.819

Notes. γ = Goodman & Kruskal's gamma coefficients. RM: Rasch model; GLLRM: Graphical loglinear Rasch model

[a] The GLLRM for the Lack of Parental Stress subscale assumed that items 1 and 6, and items 1 and 17 are locally dependent and that item 1 functions differentially relative to child sample.

Additional file 1: Table 3 Item fit statistics for the Parental stress and Lack of Parental Satisfaction subscales to the final models for the behavior sample

Subscale & items	Outfit	*p*	infit	*P*	Observed γ	Expected γ	*P*
Parental Stress[a]							
3	0.865	0.760	0.858	0.279	0.660	0.586	0.411
4	1.076	0.884	1.071	0.634	0.543	0.587	0.654
9	0.804	0.312	0.910	0.291	0.621	0.565	0.419
10	0.962	0.904	1.050	0.640	0.546	0.582	0.648
12	0.678	0.047	0.849	0.057	0.667	0.555	0.102
13	1.125	0.581	1.100	0.278	0.496	0.572	0.295
14	0.989	0.985	1.028	0.887	0.549	0.593	0.680
15	1.499	0.002[+]	1.112	0.161	0.436	0.527	0.201
16	0.940	0.789	0.935	0.504	0.587	0.549	0.612
Lack of Parental Satisfaction[b]							
1	0.937	0.688	0.974	0.808	0.660	0.655	0.955
5	0.982	0.958	1.214	0.296	0.720	0.726	0.950
6	0.565	0.232	0.852	0.487	0.855	0.730	0.238
7	1.517	0.318	1.206	0.441	0.574	0.749	0.162
8	0.866	0.556	0.994	0.942	0.632	0.632	0.999
17	0.981	0.922	0.976	0.864	0.687	0.682	0.953
18	1.220	0.607	0.871	0.587	0.696	0.740	0.702

Notes. γ = Goodman & Kruskal's gamma coefficients. RM: Rasch model; GLLRM: Graphical loglinear Rasch model

[+] Benjamini-Hochberg adjusted critical levels were: 5% limit *p* = .00185, 1% limit *p* = .00037.

[a] The GLLRM for the Parental Stress subscale assumed that item 15 and 16 functions differentially relative to parent educational level.

[b] The Lack of Parental Satisfaction subscale fit a Rasch model in the behavior sample.

Additional file 1: Table 4 Item fit statistics for the Parental stress and Lack of Parental Satisfaction subscales to the final models for the ordinary sample

Subscales & items	Outfit	*p*	infit	*p*	Observed γ	Expected γ	*P*
Parental Stress[a]							
3	0.845	0.501	0.955	0.569	0.654	0.623	0.527
4	1.465	0.059	1.115	0.179	0.479	0.593	0.040[+]
9	0.948	0.584	0.931	0.162	0.566	0.505	0.195
10	1.334	0.137	1.010	0.888	0.520	0.517	0.949
12	0.862	0.147	0.897	0.036[+]	0.590	0.504	0.062
13	0.996	0.979	1.065	0.307	0.495	0.532	0.469
14	1.746	0.066	1.024	0.869	0.515	0.624	0.190
15	1.100	0.373	1.067	0.227	0.454	0.517	0.192
16	0.989	0.959	0.880	0.185	0.623	0.577	0.441
Lack of Parental Stress[b]							
1	1.229	0.609	0.992	0.970	0.834	0.866	0.564
5	0.956	0.901	0.959	0.826	0.677	0.639	0.754
6	0.787	0.723	1.141	0.571	0.898	0.875	0.732
7	0.965	0.918	1.053	0.771	0.622	0.637	0.904
8	1.173	0.343	1.009	0.952	0.553	0.568	0.847
17	0.852	0.486	0.929	0.572	0.747	0.710	0.600
18	1.075	0.894	0.944	0.849	0.691	0.659	0.849

Notes. γ = Goodman & Kruskal's gamma coefficients. RM: Rasch model; GLLRM: Graphical loglinear Rasch model;

[+] Benjamini-Hochberg adjusted critical level 5% limit was p = .00179.

[a] The GLLRM for the Parental Stress subscale assumed that items 3 and 4 were locally dependent and that item 4 functions differentially relative to child age.

[b] The GLLRM for the Lack of Parental Satisfaction subscale assumed that items 1 and 6 and items 1 and 17 were locally dependent.
