# Supplementary material for: The Parental Stress Scale revisited: Rasch-based construct validity for Danish parents of children 2–18 years old with and without behavioral problems
Source: Health Qual Life Outcomes. 2020 Aug 17;18:281. doi: 10.1186/s12955-020-01495-w (PMC7430114; doi:10.1186/s12955-020-01495-w)
Supplement: Supplementary file 2 — Additional file 2. Appendix with conversion tables. [file 12955_2020_1495_MOESM2_ESM.docx]

# Additional file 2: Appendix

The appendix provides information on both the estimated person parameters, which have values on interval scales (logit scores), as well as the summed scale score, which are strictly speaking ordinal scales, so that researchers and clinicians alike can decide with of these they prefer to use. First the tables make it possible to convert the summed scale scores estimated person parameters. Second the tables provide information on the standard errors of measurement (SEMs) are provided for all score and person parameter values, as well as information on the amount of bias at different levels of the person parameter scale. When a subscale is affected by DIF, the tables also provide 1) DIF-adjusted person parameter values for the different subgroups, and 2) the necessary information for equating the sum scores to account for the DIF for subgroups relative to the reference group (always the first group). We first provide conversion tables for each of the subscales in the joined total sample, and then in the separate subsamples.

## Conversion tables for the total sample

Additional file 2: Table 1 Conversion of raw scores to weighted maximum likelihood estimates of person parameters and adjustment of the raw score for DIF relative to sample and parent education, for the Parental Stress subscale in the total sample

| **REFERENCE GROUP** | | | | |  |  |  |  |  |  |
| --- | --- | --- | --- | --- | --- | --- | --- | --- | --- | --- |
| **Ordinary sample, parent with secondary or less education** | | | | |  | **Behavior sample, parent with secondary or less education** | | | | |
| Score | p.param | p.param SEM | bias | score SEM |  | DIF-adjusted score | p.param | p.param SEM | bias | score SEM |
| 0.00 | -3.993 | 0.854 | 0.494 | 0.63 |  | 0.00 | -4.112 | 0.838 | 0.492 | 0.65 |
| 1.00 | -2.762 | 0.907 | 0.072 | 0.97 |  | 0.86 | -2.906 | 0.861 | 0.066 | 1.01 |
| 2.00 | -1.977 | 0.898 | 0.024 | 1.10 |  | 1.71 | -2.199 | 0.841 | 0.014 | 1.18 |
| 3.00 | -1.215 | 0.846 | 0.007 | 1.16 |  | 2.53 | -1.584 | 0.797 | 0.006 | 1.25 |
| 4.00 | -0.504 | 0.820 | -0.007 | 1.21 |  | 3.36 | -0.992 | 0.782 | 0.001 | 1.27 |
| 5.00 | 0.114 | 0.823 | -0.005 | 1.23 |  | 4.25 | -0.411 | 0.807 | 0.002 | 1.26 |
| 6.00 | 0.721 | 0.859 | 0.004 | 1.19 |  | 5.28 | 0.202 | 0.871 | 0.008 | 1.18 |
| 7.00 | 1.434 | 0.941 | 0.003 | 1.07 |  | 6.47 | 0.953 | 0.979 | 0.008 | 1.04 |
| 8.00 | 2.460 | 1.055 | -0.060 | 0.86 |  | 7.79 | 2.142 | 1.134 | -0.070 | 0.81 |
| 9.00 | 4.204 | 1.050 | -0.583 | 0.54 |  | 9.00 | 4.136 | 1.147 | -0.622 | 0.51 |
| **Ordinary sample, parent with tertiary education** | | | | |  | **Behavior sample, parent with tertiary education** | | | | |
| DIF-adjusted score | p.param | p.param SEM | bias | score SEM |  | DIF-adjusted score | p.param | p.param SEM | bias | score SEM |
| 0.00 | -3.573 | 0.839 | 0.490 | 0.64 |  | 0.00 | -3.759 | 0.827 | 0.489 | 0.65 |
| 1.34 | -2.369 | 0.882 | 0.068 | 0.99 |  | 1.13 | -2.571 | 0.839 | 0.060 | 1.04 |
| 2.42 | -1.637 | 0.879 | 0.022 | 1.13 |  | 2.05 | -1.906 | 0.826 | 0.010 | 1.21 |
| 3.42 | -0.928 | 0.839 | 0.010 | 1.17 |  | 2.88 | -1.338 | 0.795 | 0.008 | 1.27 |
| 4.45 | -0.219 | 0.828 | -0.002 | 1.20 |  | 3.73 | -0.764 | 0.797 | 0.009 | 1.26 |
| 5.50 | 0.434 | 0.838 | -0.002 | 1.20 |  | 4.71 | -0.141 | 0.839 | 0.008 | 1.20 |
| 6.50 | 1.103 | 0.870 | -0.000 | 1.16 |  | 5.86 | 0.592 | 0.909 | 0.003 | 1.10 |
| 7.40 | 1.871 | 0.940 | -0.008 | 1.06 |  | 7.06 | 1.526 | 1.009 | -0.015 | 0.98 |
| 8.21 | 2.836 | 1.012 | -0.064 | 0.89 |  | 8.09 | 2.676 | 1.089 | -0.075 | 0.84 |
| 9.00 | 4.377 | 0.975 | -0.549 | 0.57 |  | 9.00 | 4.311 | 1.016 | -0.563 | 0.55 |

Notes. p.param: Person parameter; SEM: standard error of measurement

Additional file 2: Table 2 Conversion of raw scores to weighted maximum likelihood estimates of person parameters and adjustment of the raw score for DIF relative to sample, child age and parent education, for the Lack of Parental Satisfaction subscale in the total sample

| **REFERENCE GROUP** | | | | |  |  |  | | | |
| --- | --- | --- | --- | --- | --- | --- | --- | --- | --- | --- |
| **Ordinary sample** | | | | |  | **Behavior sample** | | | | |
| score | p.param | p.param SEM | Bias | score SEM |  | DIF-adjusted score | p.param | p.param SEM | bias | score SEM |
| 0.00 | -4.090 | 1.169 | 0.640 | 0.51 |  | 0.00 | -4.115 | 0.986 | 0.562 | 0.56 |
| 1.00 | -2.034 | 1.034 | 0.051 | 0.91 |  | 0.79 | -2.511 | 0.958 | 0.036 | 0.96 |
| 2.00 | -1.170 | 0.882 | -0.018 | 1.19 |  | 1.46 | -1.757 | 0.904 | 0.001 | 1.14 |
| 3.00 | -0.652 | 0.820 | -0.007 | 1.30 |  | 2.31 | -1.146 | 0.861 | 0.011 | 1.18 |
| 4.00 | -0.164 | 0.805 | 0.009 | 1.28 |  | 3.52 | -0.451 | 0.865 | 0.004 | 1.13 |
| 5.00 | 0.430 | 0.851 | 0.003 | 1.17 |  | 4.83 | 0.358 | 0.932 | -0.016 | 1.06 |
| 6.00 | 1.257 | 0.925 | -0.062 | 0.96 |  | 5.97 | 1.252 | 0.973 | -0.068 | 0.92 |
| 7.00 | 2.651 | 0.917 | -0.528 | 0.60 |  | 7.00 | 2.654 | 0.922 | -0.525 | 0.59 |

Notes. p.param: Person parameter; SEM: standard error of measurement

## Conversion tables for the behavior sample

Additional file 2: Table 3 Conversion of raw scores to weighted maximum likelihood estimates of person parameters and adjustment of the raw score for DIF relative to parent education, for the Parental Stress subscale in the behavior sample

|  | **REFERENCE GROUP** | | | |  |  | | | | |
| --- | --- | --- | --- | --- | --- | --- | --- | --- | --- | --- |
|  | **Secondary or less education** | | | |  | **Tertiary education** | | | | |
| score | p.param | p.param SEM | Bias | score SEM |  | DIF-equated score | p.param | p.param SEM | bias | score SEM |
| 0.00 | -3.891 | 0.903 | 0.521 | 0.61 |  | 0.00 | -3.871 | 0.911 | 0.522 | 0.60 |
| 1.00 | -2.529 | 0.927 | 0.062 | 0.96 |  | 1.04 | -2.487 | 0.946 | 0.062 | 0.94 |
| 2.00 | -1.716 | 0.883 | 0.009 | 1.13 |  | 2.11 | -1.646 | 0.912 | 0.011 | 1.09 |
| 3.00 | -1.045 | 0.826 | 0.003 | 1.21 |  | 3.21 | -0.924 | 0.872 | 0.007 | 1.15 |
| 4.00 | -0.428 | 0.803 | 0.001 | 1.25 |  | 4.37 | -0.222 | 0.869 | 0.008 | 1.15 |
| 5.00 | 0.177 | 0.818 | 0.002 | 1.23 |  | 5.55 | 0.530 | 0.895 | 0.005 | 1.11 |
| 6.00 | 0.819 | 0.875 | 0.005 | 1.17 |  | 6.66 | 1.384 | 0.938 | -0.006 | 1.05 |
| 7.00 | 1.580 | 0.975 | 0.004 | 1.04 |  | 7.57 | 2.315 | 1.000 | -0.019 | 0.99 |
| 8.00 | 2.718 | 1.110 | -0.066 | 0.82 |  | 8.30 | 3.336 | 1.039 | -0.068 | 0.87 |
| 9.00 | 4.612 | 1.109 | -0.606 | 0.52 |  | 9.00 | 4.864 | 0.972 | -0.545 | 0.57 |

Notes. p.param = Person parameter. SEM = standard error of measurement

Additional file 2: Table 4 Conversion of raw scores to weighted maximum likelihood estimates of person parameters for the Lack of Parental Satisfaction scale in the behavior sample

| **Behavior sample** | | | | |
| --- | --- | --- | --- | --- |
| score | p.param | p.param SEM | bias | score SEM |
| 0.00 | -3.474 | 0.964 | 0.545 | 0.57 |
| 1.00 | -1.960 | 0.999 | 0.063 | 0.90 |
| 2.00 | -1.020 | 0.935 | 0.007 | 1.06 |
| 3.00 | -0.261 | 0.872 | -0.003 | 1.15 |
| 4.00 | 0.420 | 0.859 | -0.006 | 1.17 |
| 5.00 | 1.096 | 0.891 | -0.009 | 1.12 |
| 6.00 | 1.883 | 0.971 | -0.059 | 0.96 |
| 7.00 | 3.215 | 0.888 | -0.513 | 0.61 |

Notes. p.param = Person parameter. SEM = standard error of measurement

## Conversion tables for the ordinary sample

Additional file 2: Table 5 Conversion of raw scores to weighted maximum likelihood estimates of person parameters and adjustment of the raw score for DIF relative to child age, for the Parental Stress scale in the ordinary sample

|  | **REFERENCE GROUP** | | | |  |  | | | | |  |  | | | | |
| --- | --- | --- | --- | --- | --- | --- | --- | --- | --- | --- | --- | --- | --- | --- | --- | --- |
|  | **Child age 2-5 years** | | | |  | **Child age 6-10 years** | | | | |  | **Child age 11-18 years** | | | | |
| score | p.param | p.param SEM | Bias | score SEM |  | DIF-  equated  score | p.param | p.param SEM | bias | score SEM |  | DIF-  equated  score | p.param | p.param SEM | bias | score SEM |
| 0.00 | -3.864 | 0.862 | 0.505 | 0.63 |  | 0.00 | -3.978 | 0.841 | 0.492 | 0.64 |  | 0.00 | -4.208 | 0.842 | 0.490 | 0.64 |
| 1.00 | -2.590 | 0.881 | 0.057 | 1.00 |  | 0.88 | -2.760 | 0.878 | 0.063 | 1.00 |  | 0.73 | -3.001 | 0.898 | 0.071 | 0.98 |
| 2.00 | -1.878 | 0.866 | 0.013 | 1.16 |  | 1.81 | -2.046 | 0.878 | 0.020 | 1.14 |  | 1.59 | -2.245 | 0.906 | 0.027 | 1.09 |
| 3.00 | -1.246 | 0.827 | 0.012 | 1.21 |  | 2.84 | -1.368 | 0.846 | 0.014 | 1.17 |  | 2.67 | -1.478 | 0.876 | 0.014 | 1.12 |
| 4.00 | -0.583 | 0.816 | 0.004 | 1.21 |  | 3.90 | -0.636 | 0.839 | 0.000 | 1.17 |  | 3.82 | -0.699 | 0.866 | -0.004 | 1.14 |
| 5.00 | 0.093 | 0.837 | -0.003 | 1.19 |  | 4.95 | 0.073 | 0.854 | -0.005 | 1.17 |  | 4.92 | -0.061 | 0.869 | -0.007 | 1.16 |
| 6.00 | 0.783 | 0.878 | -0.003 | 1.15 |  | 5.98 | 0.771 | 0.886 | -0.003 | 1.14 |  | 5.96 | 0.765 | 0.892 | -0.002 | 1.13 |
| 7.00 | 1.553 | 0.949 | -0.007 | 1.05 |  | 6.99 | 1.548 | 0.952 | -0.007 | 1.05 |  | 6.99 | 1.545 | 0.954 | -0.006 | 1.05 |
| 8.00 | 2.537 | 1.025 | -0.063 | 0.88 |  | 8.00 | 2.533 | 1.025 | -0.063 | 0.88 |  | 8.00 | 2.532 | 1.025 | -0.063 | 0.88 |
| 9.00 | 4.121 | 0.991 | -0.556 | 0.56 |  | 9.00 | 4.117 | 0.991 | -0.557 | 0.56 |  | 9.00 | 4.114 | 0.991 | -0.557 | 0.56 |

Notes. p.param = Person parameter. SEM = standard error of measurement

Additional file 2: Table 6 Conversion of raw scores to weighted maximum likelihood estimates of person parameters for the Lack of Parental Satisfaction scale in the ordinary sample

| **Ordinary sample** | | | | |
| --- | --- | --- | --- | --- |
| score | p.param | p.param SEM | bias | score SEM |
| 0.00 | -4.428 | 1.256 | 0.679 | 0.49 |
| 1.00 | -2.151 | 1.019 | 0.047 | 0.94 |
| 2.00 | -1.415 | 0.877 | -0.011 | 1.22 |
| 3.00 | -0.950 | 0.828 | 0.005 | 1.31 |
| 4.00 | -0.461 | 0.821 | 0.019 | 1.25 |
| 5.00 | 0.260 | 0.899 | -0.004 | 1.08 |
| 6.00 | 1.249 | 1.002 | -0.076 | 0.90 |
| 7.00 | 2.737 | 0.960 | -0.543 | 0.58 |

Notes. p.param = Person parameter. SEM = standard error of measurement
