# Supplementary material for: The Parental Stress Scale revisited: Rasch-based construct validity for Danish parents of children 2–18 years old with and without behavioral problems
Source: Health Qual Life Outcomes. 2020 Aug 17;18:281. doi: 10.1186/s12955-020-01495-w (PMC7430114; doi:10.1186/s12955-020-01495-w)
Supplement: Supplementary file 3 — Additional file 3. Contains consistency tables with inter-item correlations and item-restscore correlations for each subscale in each subsample. [file 12955_2020_1495_MOESM3_ESM.pdf]

Additional file 3:

Consistency tables with marginal associations among items as well as between items and restscores (i.e. item correlation with the scale score with this item subtracted). Correlations are gamma correlations.

Lack of parental satisfaction subscale

Behavior sample

items			A	E	F	G	H	Q	R	rest score
A	pss1	Gamma		0.605	0.880	0.274	0.657	0.754	0.831	0.660
		p		0.018	0.000	0.246	0.000	0.000	0.003	0.000
E	pss5	Gamma	0.605		0.773	0.554	0.758	0.587	0.632	0.720
		p	0.018		0.024	0.160	0.001	0.037	0.091	0.000
F	pss6	Gamma	0.880	0.773		0.843	0.737	0.771	0.845	0.855
		p	0.000	0.024		0.031	0.002	0.006	0.018	0.000
G	pss7	Gamma	0.274	0.554	0.843		0.788	0.470	0.658	0.574
		p	0.246	0.160	0.031		0.006	0.141	0.132	0.017
H	pss8	Gamma	0.657	0.758	0.737	0.788		0.663	0.652	0.632
		p	0.000	0.001	0.002	0.006		0.000	0.011	0.000
Q	pss17	Gamma	0.754	0.587	0.771	0.470	0.663		0.897	0.687
		p	0.000	0.037	0.006	0.141	0.000		0.002	0.000
R	pss18	Gamma	0.831	0.632	0.845	0.658	0.652	0.897		0.696
		p	0.003	0.091	0.018	0.132	0.011	0.002		0.004

Ordinary sample

	items		A	E	F	G	H	Q	R	rest score
A	pss1	Gamma		0.899	0.976	0.779	0.595	0.941	0.877	0.834
		p		0.002	0.000	0.015	0.000	0.000	0.030	0.000
E	pss5	Gamma	0.899		0.893	0.679	0.376	0.833	0.792	0.677
		p	0.002		0.010	0.081	0.050	0.002	0.105	0.001
F	pss6	Gamma	0.976	0.893		0.695	0.700	0.904	0.934	0.898
		p	0.000	0.010		0.078	0.001	0.000	0.026	0.000
G	pss7	Gamma	0.779	0.679	0.695		0.657	0.673	0.544	0.622
		p	0.015	0.081	0.078		0.001	0.017	0.242	0.001
H	pss8	Gamma	0.595	0.376	0.700	0.657		0.638	0.488	0.553
		p	0.000	0.050	0.001	0.001		0.000	0.074	0.000
Q	pss17	Gamma	0.941	0.833	0.904	0.673	0.638		0.852	0.747
		p	0.000	0.002	0.000	0.017	0.000		0.019	0.000
R	pss18	Gamma	0.877	0.792	0.934	0.544	0.488	0.852		0.691
		p	0.030	0.105	0.026	0.242	0.074	0.019		0.016

Parental stress subscale

Behavior sample

	items		C	D	I	J	L	M	N	O	P	rest score
C	pss3	Gamma		0.635	0.685	0.553	0.748	0.548	0.305	0.453	0.678	0.660
		p		0.007	0.000	0.004	0.000	0.002	0.159	0.006	0.000	0.000
D	pss4	Gamma	0.635		0.480	0.396	0.585	0.525	0.511	0.383	0.336	0.543
		p	0.007		0.009	0.048	0.001	0.006	0.056	0.029	0.062	0.000
I	pss9	Gamma	0.685	0.480		0.617	0.703	0.595	0.624	0.300	0.557	0.621
		p	0.000	0.009		0.000	0.000	0.000	0.002	0.012	0.000	0.000
J	pss10	Gamma	0.553	0.396	0.617		0.659	0.399	0.750	0.348	0.413	0.546
		p	0.004	0.048	0.000		0.000	0.007	0.001	0.012	0.006	0.000
L	pss12	Gamma	0.748	0.585	0.703	0.659		0.443	0.624	0.528	0.592	0.667
		p	0.000	0.001	0.000	0.000		0.000	0.001	0.000	0.000	0.000
M	pss13	Gamma	0.548	0.525	0.595	0.399	0.443		0.684	0.229	0.482	0.496
		p	0.002	0.006	0.000	0.007	0.000		0.001	0.052	0.000	0.000
N	pss14	Gamma	0.305	0.511	0.624	0.750	0.624	0.684		0.226	0.557	0.549
		p	0.159	0.056	0.002	0.001	0.001	0.001		0.153	0.007	0.000
O	pss15	Gamma	0.453	0.383	0.300	0.348	0.528	0.229	0.226		0.581	0.436
		p	0.006	0.029	0.012	0.012	0.000	0.052	0.153		0.000	0.000
P	pss16	Gamma	0.678	0.336	0.557	0.413	0.592	0.482	0.557	0.581		0.587
		p	0.000	0.062	0.000	0.006	0.000	0.000	0.007	0.000		0.000

ordinary sample

items			C	D	I	J	L	M	N	O	P	rest score
C	pss3	Gamma		0.754	0.613	0.516	0.668	0.622	0.139	0.478	0.379	0.654
		p		0.000	0.000	0.000	0.000	0.000	0.244	0.000	0.002	0.000
D	pss4	Gamma	0.754		0.304	0.261	0.429	0.453	0.606	0.482	0.213	0.479
		p	0.000		0.002	0.026	0.000	0.000	0.001	0.000	0.074	0.000
I	pss9	Gamma	0.613	0.304		0.639	0.590	0.504	0.360	0.479	0.506	0.566
		p	0.000	0.002		0.000	0.000	0.000	0.017	0.000	0.000	0.000
J	pss10	Gamma	0.516	0.261	0.639		0.594	0.467	0.123	0.306	0.727	0.520
		p	0.000	0.026	0.000		0.000	0.000	0.283	0.003	0.000	0.000
L	pss12	Gamma	0.668	0.429	0.590	0.594		0.507	0.470	0.374	0.667	0.590
		p	0.000	0.000	0.000	0.000		0.000	0.002	0.000	0.000	0.000
M	pss13	Gamma	0.622	0.453	0.504	0.467	0.507		0.431	0.330	0.547	0.495
		p	0.000	0.000	0.000	0.000	0.000		0.010	0.000	0.000	0.000
N	pss14	Gamma	0.139	0.606	0.360	0.123	0.470	0.431		0.618	0.837	0.515
		p	0.244	0.001	0.017	0.283	0.002	0.010		0.000	0.000	0.000
O	pss15	Gamma	0.478	0.482	0.479	0.306	0.374	0.330	0.618		0.657	0.454
		p	0.000	0.000	0.000	0.003	0.000	0.000	0.000		0.000	0.000
P	pss16	Gamma	0.379	0.213	0.506	0.727	0.667	0.547	0.837	0.657		0.623
		p	0.002	0.074	0.000	0.000	0.000	0.000	0.000	0.000		0.000
